# Supplementary material for: TMEM135 links peroxisomes to the regulation of brown fat mitochondrial fission and energy homeostasis
Source: Nat Commun. 2023 Sep 29;14:6099. doi: 10.1038/s41467-023-41849-8 (PMC10541902; doi:10.1038/s41467-023-41849-8)
Supplement: Supplementary file 1 — Supplementary Information [file 41467_2023_41849_MOESM1_ESM.pdf]

Supplementary Information

TMEM135 Links Peroxisomes to the Regulation of Brown Fat Mitochondrial Fission and Energy Homeostasis

Donghua Hu, Min Tan[1], Dongliang Lu, Brian Kleiboeker, Xuejing Liu, Hongsuk Park, Alexxai V. Kravitz, Kooresh I. Shoghi, Yu-Hua Tseng, Babak Razani, Akihiro Ikeda, and Irfan J. Lodhi

Supplementary information includes seven Supplementary Figures and one Supplementary Table.

Supplementary Fig. 1. Effect of Pex16 deficiency on mitochondrial respiration and mitophagy in mouse brown adipocytes.

Supplementary Fig. 2. TMEM135 is present in peroxisomes and mitochondria.

Supplementary Fig. 3. TMEM135 gene expression increases during BAT SVF cell differentiation and after NE treatment.

Supplementary Fig.4. Metabolic phenotyping of *Tmem135-AKO* mice.

Supplementary Fig. 5. Metabolic phenotyping of *Tmem135*^{TG} mice.

Supplementary Fig. 6. Overexpression of TMEM135 in *Pex16-AKO* mice rescues cold tolerance and mtDNA copy number.

Supplementary Fig. 7. Gene expression analysis and effect of TMEM135 KD on mitochondrial morphology in different cell types**.**

Supplementary Table 1. Oligonucleotide sequences

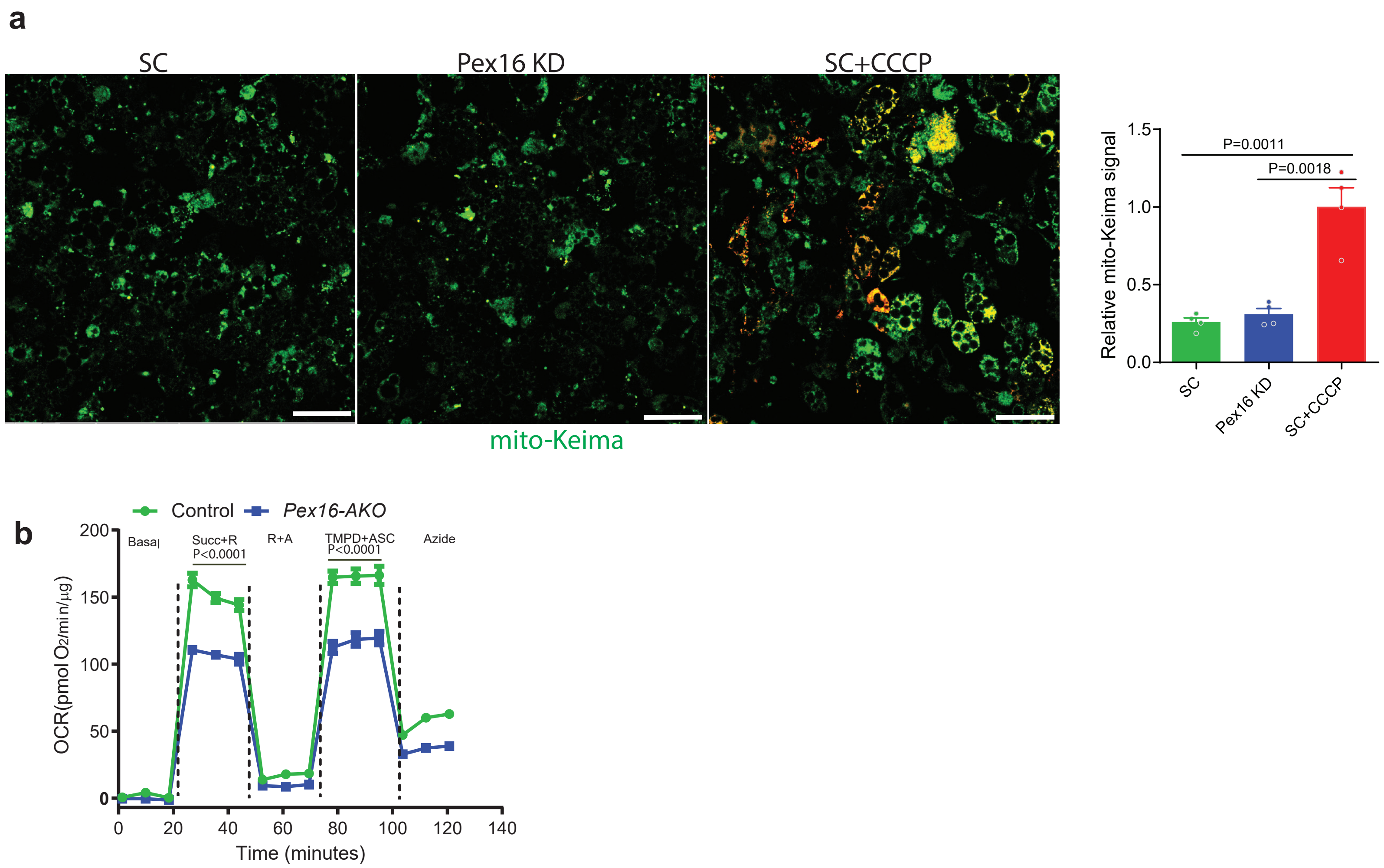

Supplementary Fig. 1. Effect of Pex16 deficiency on mitochondrial respiration and mitophagy in mouse brown adipocytes. (**a**) Confocal imaging of mitophagy using mito-Keima in brown adipocytes treated with scrambled (SC) (n=4) or Pex16 shRNA (n=4). Carbonyl cyanide m-chlorophenylhydrazone (CCCP) was used as a positive control (n=4) to induce mitophagy. Plot shows quantification of mito-Keima signal. Scale bar: 50 μm. (**b**) OCR measured using a Seahorse XF24 Extracellular Flux Analyzer in frozen BAT isolated from control (n=5) and Pex16-AKO (n=5) mice subjected to 3 day cold exposure. Succ+R, Succinate + Rotenone; R+A, Rotenone + antimycin A; TMPD+ASC, N,N,N',N'-tetramethyl-p-phenylenediamine + Ascorbic acid. Data are presented as mean ± SEM; statistical significance was determined by Student's t test (**a**) or 2-way ANOVA with Bonferroni's post hoc test (**b**).

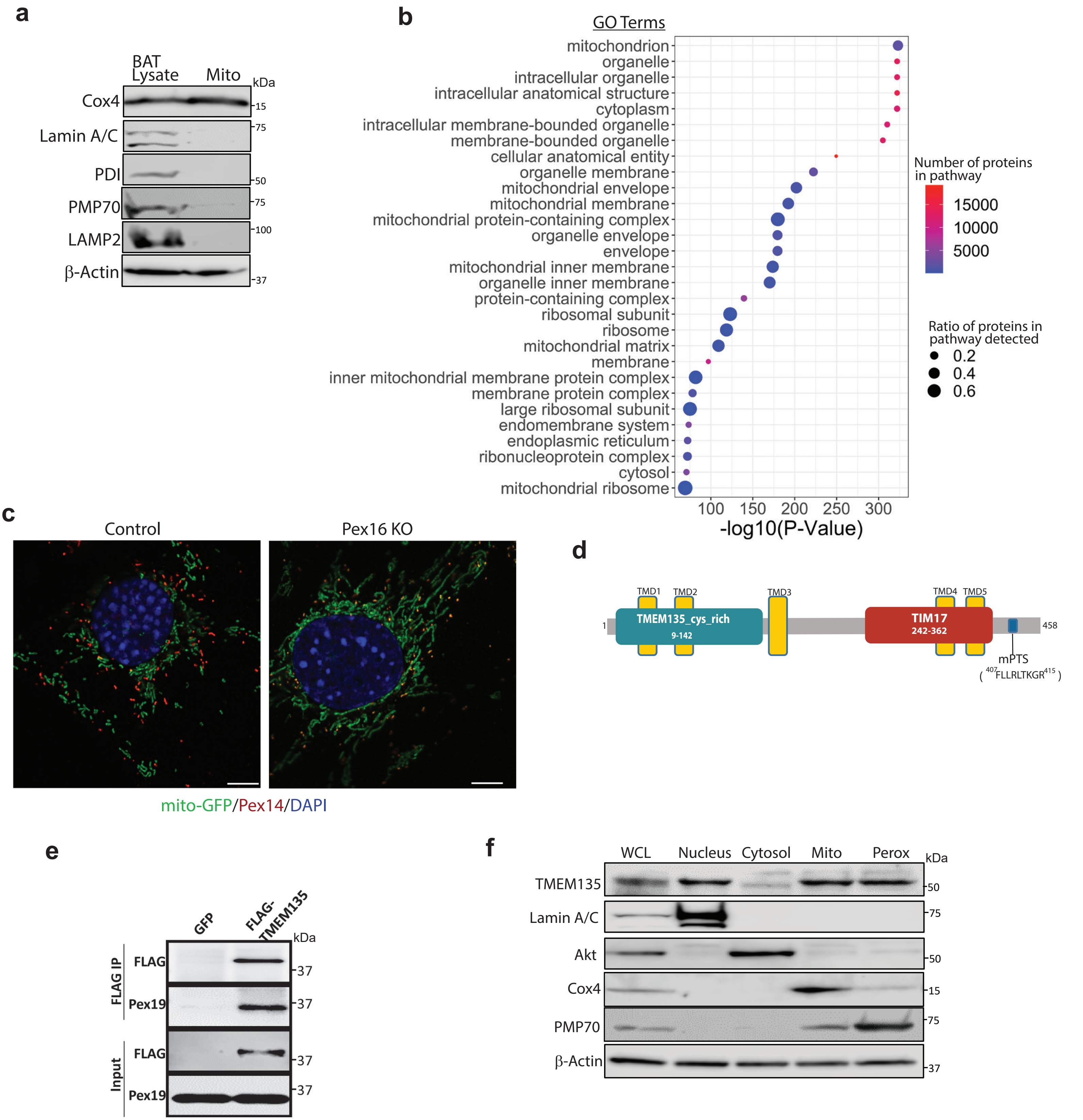

Supplementary Fig. 2. TMEM135 is present in peroxisomes and mitochondria.
(**a**) Western blot analysis of whole BAT lysate and mitochondrial fraction from brown adipose of wild-type C57BL/6J mice. (**b**) Gene Ontology (GO) terms (cellular component ontology) for unchanged proteins identified by mass spectrometry-based proteomics in mitochondria isolated from BAT of control and Pex16-AKO mice. (**c**) Immunofluorescence analysis in control and Pex16-AKO brown adipocytes expressing mito-GFP stained with an antibody against Pex14. Scale bar: 5 μm. (**d**) A model of TMEM135 domain architecture showing that the protein contains an N-terminal TMEM135_Cys_rich domain and a C-terminal TIM17 domain. (**e**) Co-immunoprecipitation of endogenous Pex19 with FLAG-TMEM135 in HEK293 cells. (**f**) Western blot analysis of TMEM135 and organelle markers in whole cell lysates (WCL) and various subcellular fractions of differentiated brown pre-adipocytes from the wild-type C57BL/6J mice.

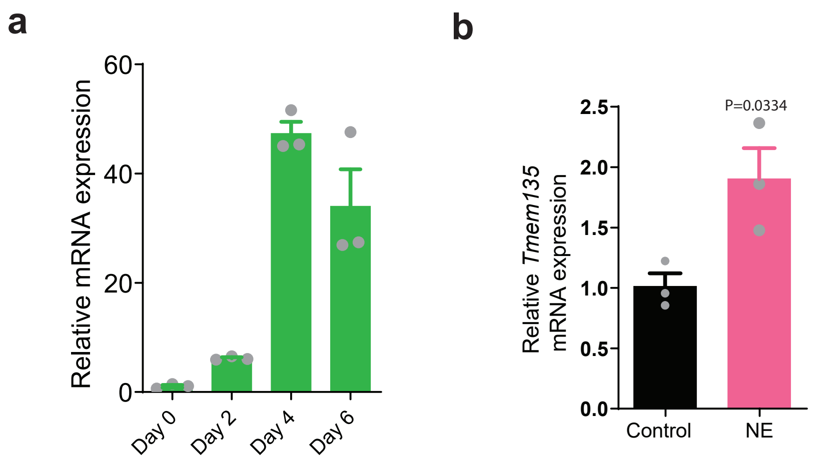

Supplementary Fig. 3. TMEM135 gene expression increases during BAT SVF cell differentiation and after NE treatment. (**a**) qPCR analysis of *Tmem135* expression during adipogenesis in mouse BAT SVF cells; n=3. (**b**) qPCR analysis of *Tmem135* expression in brown adipocytes treated with NE (1μM) for 2 hours; n=3. Data are presented as mean ± SEM; statistical significance was determined by two-tailed unpaired Student's t test (**b**).

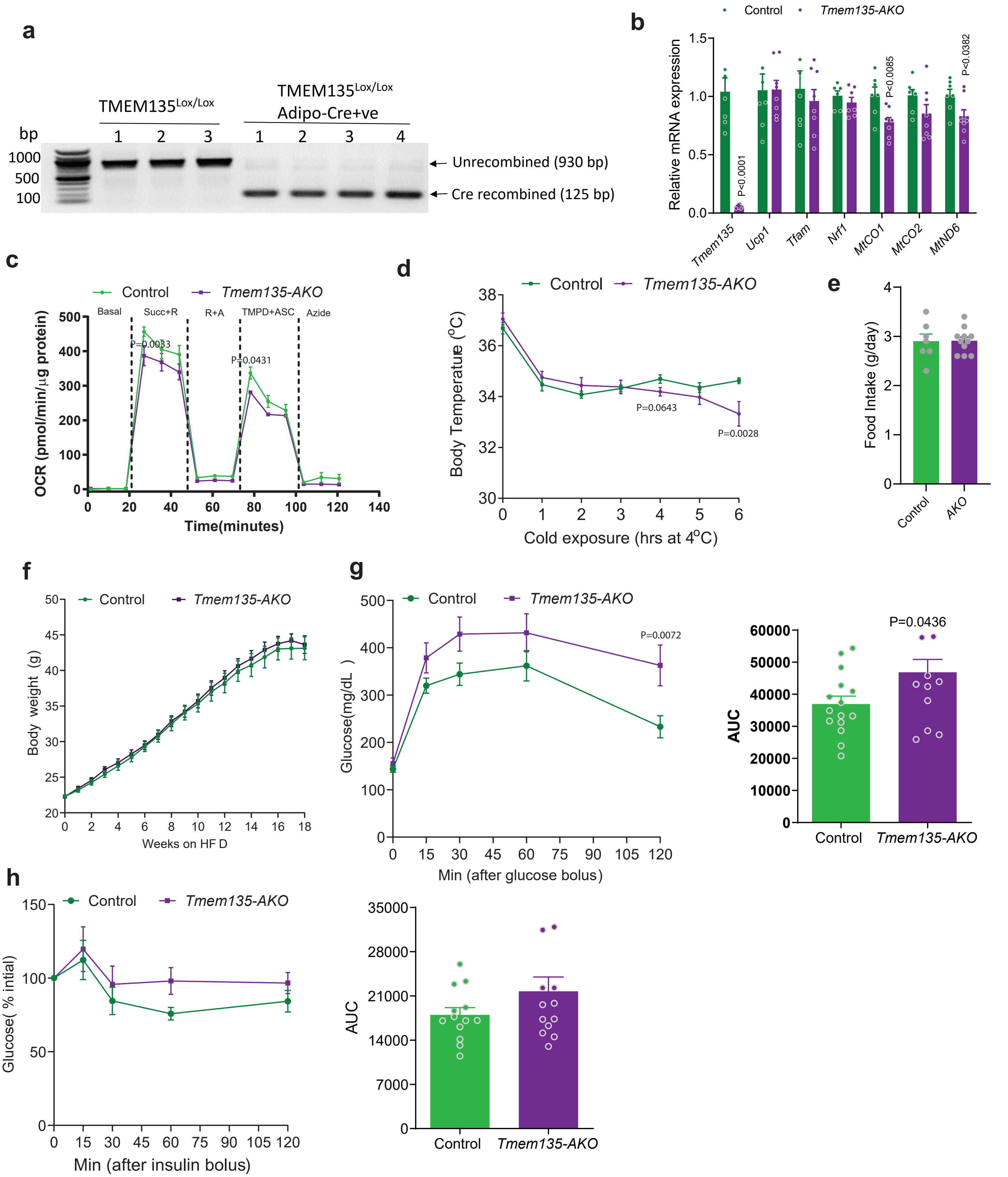

Supplementary Fig.4. Metabolic phenotyping of *Tmem135-AKO* mice.
(**a**) Confirmation of Cre-mediated recombination of the floxed alleles in *Tmem135-AKO* mice. (**b**) qPCR analysis of mitochondria-encoded genes in control (n=7) and *Tmem135-AKO* (n=8) mice. (**c**) OCR measured using a Seahorse XF24 Extracellular Flux Analyzer in frozen BAT isolated from control (n=5) and *Tmem135-AKO* (n=5) mice subjected to 7 day cold exposure; Succ+R, Succinate + Rotenone; R+A, Rotenone + antimycin A; TMPD+ASC, N,N,N',N'-tetramethyl-p-phenylenediamine + Ascorbic acid.

(**d**) Body temperature of male *Tmem135-AKO* (n=10) and control (n=17) mice subjected to a 6-hour cold challenge. (**e**) Food intake of female HFD-fed *Tmem135-AKO* (n=11) and control (n=7) mice. (**f**) Body weight of male HFD-fed *Tmem135-AKO* (n=15) and control (n=16) mice. (**g**) Glucose tolerance testing and AUC in male HFD-fed *Tmem135-AKO* (n=13) and control (n=15) mice. (**h**) Insulin tolerance testing and AUC in male HFD-fed *Tmem135-AKO* (n=13) and control (n=13) mice. Data are presented as mean ± SEM; statistical significance was determined by two-tailed unpaired Student's t test (**b, d, e, f, g** and **h**) or 2-way ANOVA with Bonferroni's post hoc test (**c, g,** and **h**).

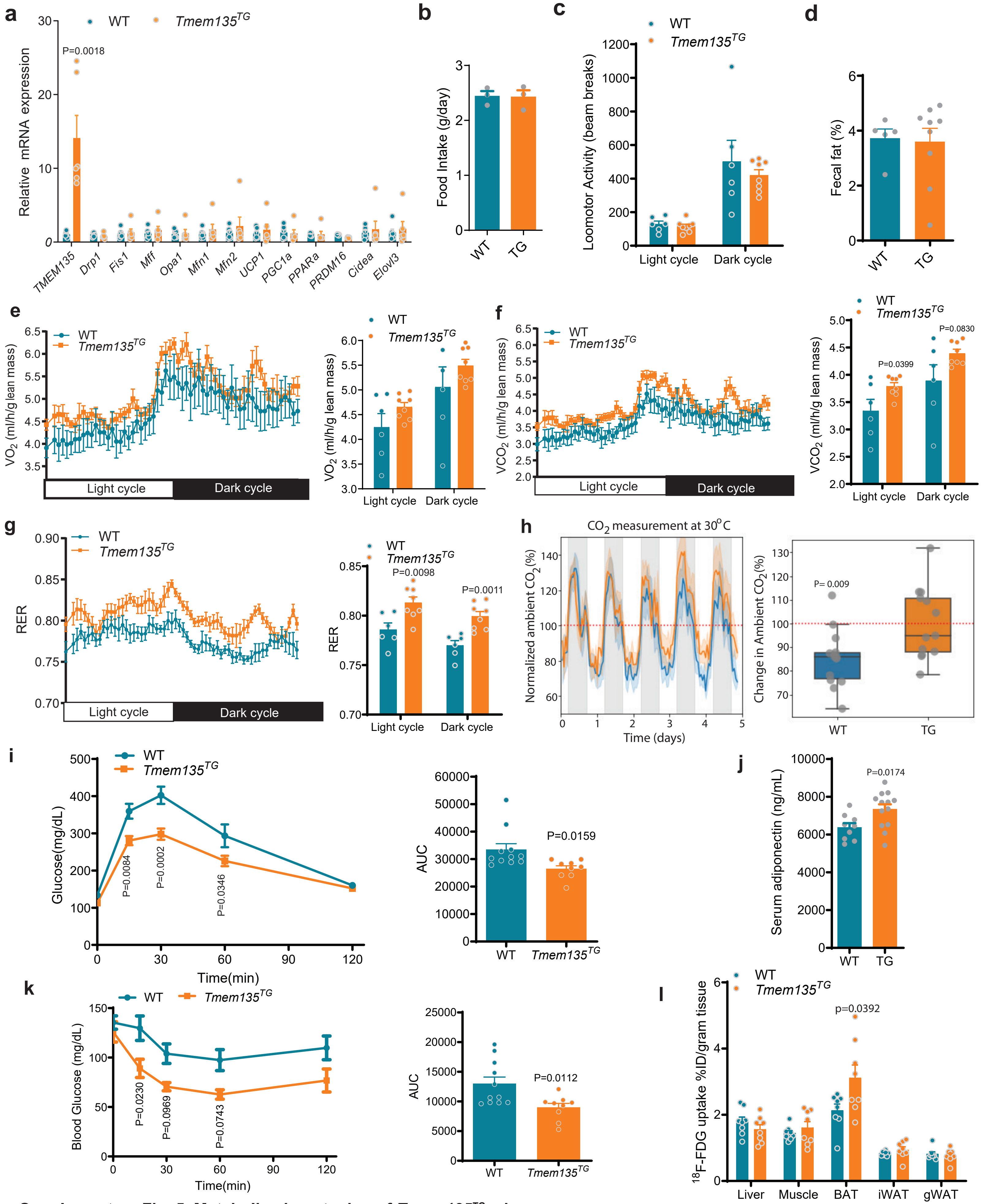

Supplementary Fig. 5. Metabolic phenotyping of *Tmem135*^{TG} mice.
(**a**) qPCR analysis of mitochondrial dynamics and thermogenic genes in BAT of *Tmem135*^{TG} (n=6) and WT (n=6) mice. (**b**) Food

intake of male *Tmem135*^{TG} (n=3) and WT (n=3) mice fed a high fat diet. (**c**) Locomotor activity in male HFD-fed *Tmem135*^{TG} (n=8) and WT (n=6) mice. (**d**) Fecal fat of male HFD-fed *Tmem135*^{TG} mice fed a high fat diet. These mice were placed in metabolic cages for 48 hours with 12-hour light/dark cycles; WT n=5; Tmem135^{TG} n=9. (**e**)-(**g**) VO_2, VCO_2, and respiratory exchange ratio (RER) of *Tmem135*^{TG} (n=8) and WT (n=6) mice fed a HFD for 4 weeks, prior to significant difference in body weight. (**h**) Change of carbon dioxide generation using an in-cage CO_2 monitor in *Tmem135*^{TG} (n=14) and WT (n=13) mice maintained at thermoneutrality. (**i**) Glucose tolerance testing and AUC of female HFD-fed *Tmem135*^{TG} (n=9) and WT (n=11) mice. (**j**) Fasting serum adiponectin of male HFD-fed *Tmem135*^{TG} (n=13) and WT (n=9) mice. (**k**) Insulin tolerance testing and AUC of female HFD-fed Tmem135^{TG} (n=9) and WT (n=11) mice. (**l**) Quantification of ^{18}F-FDG uptake in indicated tissues from male Tmem135^{TG} (n=8) and WT (n=8) mice fed with a high fat diet for 14 weeks. Data are presented as mean ± SEM; statistical significance was determined by two-tailed unpaired Student's t test (**a-d, h, l, j, k** and **l**) or 2-way ANOVA with Tukey's honest significant difference post hoc test (**e-g**) or Bonferroni's post hoc test (**i** and **k**).

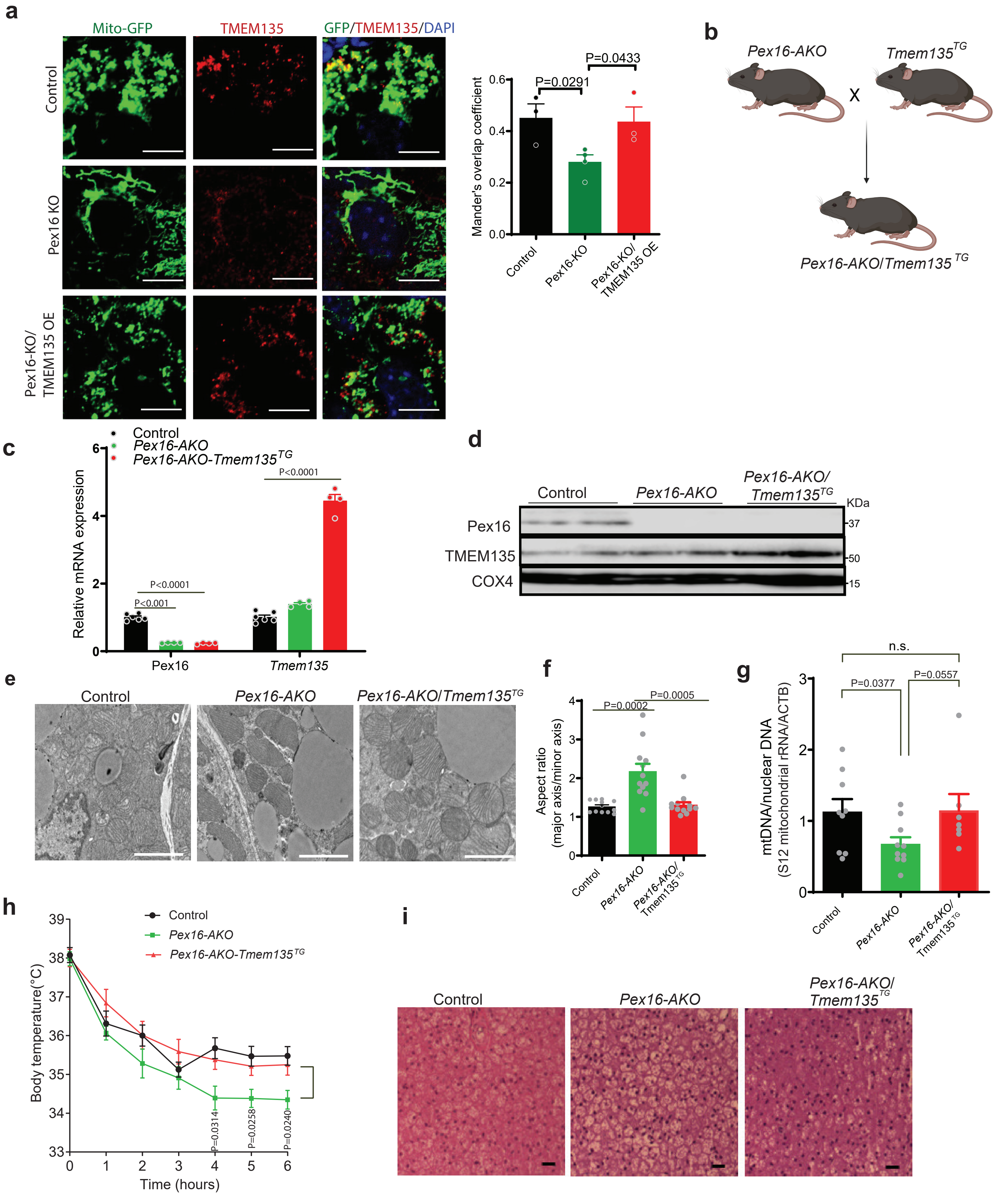

Supplementary Fig. 6. Overexpression of TMEM135 in *Pex16-AKO* mice rescues cold tolerance and mtDNA copy number. **(a)** Immunofluorescence analysis in control (*Pex16*$^{lox/lox}$), Pex16 KO (*Pex16*$^{lox/lox}$; adipo-Cre positive) and Tmem135 overexpressing Pex16 KO (Pex16 KO/TMEM135 OE) brown adipocytes stably expressing mito-GFP. Quantification of the colocalization using ImageJ. Scale bar: 10 µm. The images are representative of three independent experiments and the quantification is based on a

total of 27 control, 50 Pex16-KO cells, and 26 Pex16 KO-Tmem135 OE cells. (**b**) Simplified breeding scheme of *Pex16-AKO/ Tmem135^{TG}* double mutant mice. Created with BioRender.com. (**c**) and (**d**) qPCR (control n=6; *Pex16-AKO* n=4; *Pex16-AKO*/*Tmem135^{TG}* n= 4) and Western blot (n=2 for each group) analysis of Pex16 and TMEM135 in *Pex16-AKO*/*Tmem135^{TG}* double mutant mice. (**e**) TEM analysis of BAT from control, *Pex16-AKO* and *Pex16-AKO*/*Tmem135^{TG}* double mutant mice subjected to 3 day cold exposure. Scale bar: 1 μm. (**f**) Aspect ratio (ratio of major axis length to minor axis length) measured in BA T mitochondria. Data are based on 12 mitochondria per condition. (**g**) mtDNA copy number normalized to nuclear DNA in control, *Pex16-AKO* and *Pex16-AKO*/*Tmem135^{TG}* double mutant mice subjected to 3 day cold exposure; control n=9; *Pex16-AKO* n=10; *Pex16-AKO*/*Tmem135^{TG}* n=7. (**h**) Body temperature of control, *Pex16-AKO* and *Pex16-AKO*/*Tmem135^{TG}* double mutant mice subjected a 6-hour cold challenge; control n=9; *Pex16-AKO* n=11; *Pex16-AKO*/*Tmem135^{TG}* n= 8. (**i**) H&E staining of control, *Pex16-AKO* and *Pex16-AKO*/*Tmem135^{TG}* double mutant mice subjected to 3 days cold exposure. The images are representative of 3 mice per genotype. Scale bar: 50 μm. Data are presented as mean ± SEM; statistical significance was determined by two-tailed unpaired Student's t test (**a, c, f, g,** and **h**).

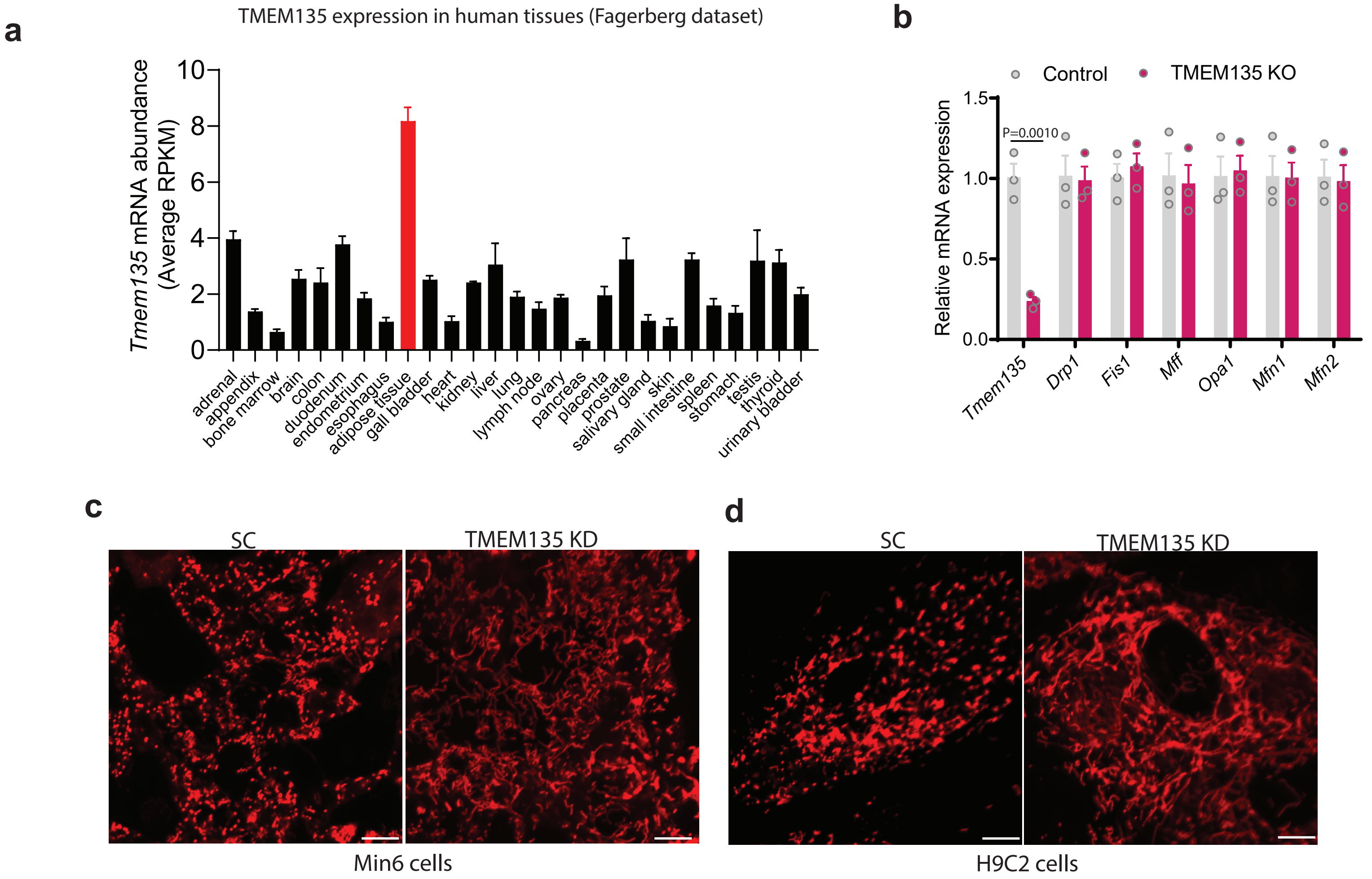

Supplementary Fig. 7. Gene expression analysis and effect of TMEM135 KD on mitochondrial morphology in different cell types.

(**a**) *Tmem135* gene expression in major human tissues, based on publicly available data (Fagerberg et al. [Ref 47]). (**b**) qPCR analysis of mitochondrial dynamics genes in control (n=3) and TMEM135 KO (n=3) human brown adipocytes. (**c**) Mitochondrial morphology of in Min6 mouse insulinoma cells treated with scrambled (SC) or TMEM135 shRNA and stained with TMRE for using confocal microscopy. Scale bar: 10 µm. (**d**) Mitochondrial morphology of in H9C2 rat cardiomyocytes treated with scrambled (SC) or TMEM135 shRNA and stained with TMRE for using confocal microscopy. Scale bar: 10 µm. Data are presented as mean ± SEM; statistical significance was determined by two-tailed unpaired Student's t test (**b**).

Supplementary Table 1. Oligonucleotide sequences.

Name	Forwad(5'-3')	Reverse (5'-3')
L-32	TTC CTG GTC CAC AAT GTC AA	GGC TTT TCG GTT CTT AGA GGA
TMEM135	AGG CTG GAA AGG TTC CCT ATT	CGC CAG TAC CAA ACA CAT CC
Pex16	CCG TTC TAT GAC CGC TTC TC	GGA GGG CAA GTA GTC CAT GA
PMP70	TCT GCC TAC TCC ATA AGC GG	CAC CAC AGC TCG CTC TTT CT
Drp1	AGA TCG TCG TAG TGG GAA CG	AGA ATG AGA GGT CTC CGG GT
Fis1	ATT TGA ATA TGC CTG GTG CC	CAT AGT CCC GCT GTT CCT CT
Opa1	TGG AAA ATG GTT CGA GAG TCA G	CAT TCC GTC TCT AGG TTA AAG CG
Mff	GGA GTT CCA AAT GCC AGT GTG ATA	TGG ATA AGG TCA AGA TCT GCT GGT
Mfn1	TGA ATA ACC GTT GGG ATG CT	GCT TCC GAC GGA CTT ACA AC
Mfn2	GTC CTG GAC GTC AAA GGG TA	ATT GAT CAC GGT GCT CTT CC
UCP1	TCA GCT GTT CAA AGC ACA CA	GTA CCA AGC TGT GCG ATG TC
PGC1α	TGT AGC GAC CAA TCG GAA AT	TGA GGA CCG CTA GCA AGT TT
PRDM16	CAG AGG TGT CAT CCC AGG AG	ACG GAT GTA CTT GAG CCA GC
PPARα	AGT TCG GGA ACA AGA CGT TG	CAG TGG GGA GAG AGG ACA GA
Cidea	CAGTGATTTAAGAGACGCGG	TCTGCAATCCCATGAATGTC
Elovl3	CTT AAG GCC CTT TTT GGA GG	CCG CGT TCT CAT GTA GGT CT
GNPAT	CATGGACGTTCCTAGCTCCT	CCCACTTTTTGAGGTCTTTCG
Tfam	CCAAAAAGACCTCGTTCAGC	GACAGATTTTTCCAAGCCTCA
Nrf1	TAGTCCTGTCTGGGGAAACC	CTGGTACATGCTCACAGGGA
MtCO1	TCCTACCACCATCATTTCTCC	CTGATGCTCCTGCATGGG
MtCO2	CATCAAACCGACCAGGGTT	AATTATTGAAGCAGATCAGTTTTCG
MtND6	CCAACATAACTCCAACATCATCA	GTATTGGGGGTGATTATAGAGGTTT
Mito-DNA	TTA AGA CAC CTT GCC TAG CCA CAC	CGG TGG CTG GCA CGA AAT T
Nucler-DNA	ATG ACG ATA TCG CTG CGC TG	TCA CTT ACC TGG TGC CTA GGG C
huL-32	AAG TTC CTG GTC CAC AAC GTC AA	CAG CTC TTT CCA CGA CTT T
huTMEM135	CTC CTC TGT ACT TGA TTG CAG C	TGA AGC GGA TTG TAG GAT CTC A
hDrp1	CTG CCT CAA ATC GTC GTA GTG	GAG GTC TCC GGG TGA CAA TTC
hFis1	GTC CAA GAG CAC GCA GTT TG	ATG CCT TTA CGG ATG TCA TCA TT
hOpa1	TGT GAG GTC TGC CAG TCT TTA	TGT CCT TAA TTG GGG TCG TTG
hMff	ACTG AAG GCA TTA GTC AGC GA	TCC TGC TAC AAC AAT CCT CTC C
hMfn1	TGG CTA AGA AGG CGA TTA CTG C	TCT CCG AGA TAG CAC CTC ACC
hMfn2	CTC TCG ATG CAA CTC TAT CGT C	TCC TGT ACG TGT CTT CAA GGA A
huMito-DNA	ACA CAG CAA GAC GAG AAG ACC CTA	TAC TGC TCG GAG GTT GGG TTC T

huNucler-DNA	AAG GGC GCT TTC TCT GCA CA	CAT AGG AAT CCT TCT GAC CCA TGC
