# Supplementary material for: TMEM135 links peroxisomes to the regulation of brown fat mitochondrial fission and energy homeostasis
Source: Nat Commun. 2023 Sep 29;14:6099. doi: 10.1038/s41467-023-41849-8 (PMC10541902; doi:10.1038/s41467-023-41849-8)
Supplement: Supplementary file 3 — Description of Additional Supplementary Files [file 41467_2023_41849_MOESM3_ESM.pdf]

**Description of Additional Supplementary Files**

File Name: Supplementary Data 1
Description: Peptides identified by mass spectrometry-based proteomics in mitochondria from Pex16 KO and WT mouse BAT.
