# Supplementary material for: TMEM135 links peroxisomes to the regulation of brown fat mitochondrial fission and energy homeostasis
Source: Nat Commun. 2023 Sep 29;14:6099. doi: 10.1038/s41467-023-41849-8 (PMC10541902; doi:10.1038/s41467-023-41849-8)
Supplement: Supplementary file 5 — Reporting Summary [file 41467_2023_41849_MOESM5_ESM.pdf]

nature portfolio

Corresponding author(s): Irfan J. Lodhi

Last updated by author(s): Sep 5, 2023

Reporting Summary

Nature Portfolio wishes to improve the reproducibility of the work that we publish. This form provides structure for consistency and transparency in reporting. For further information on Nature Portfolio policies, see our Editorial Policies and the Editorial Policy Checklist.

Statistics

For all statistical analyses, confirm that the following items are present in the figure legend, table legend, main text, or Methods section.

n/a	Confirmed	
☐	☒	The exact sample size (*n*) for each experimental group/condition, given as a discrete number and unit of measurement
☐	☒	A statement on whether measurements were taken from distinct samples or whether the same sample was measured repeatedly
☐	☒	The statistical test(s) used AND whether they are one- or two-sided<br>*Only common tests should be described solely by name; describe more complex techniques in the Methods section.*
☐	☒	A description of all covariates tested
☐	☒	A description of any assumptions or corrections, such as tests of normality and adjustment for multiple comparisons
☐	☒	A full description of the statistical parameters including central tendency (e.g. means) or other basic estimates (e.g. regression coefficient) AND variation (e.g. standard deviation) or associated estimates of uncertainty (e.g. confidence intervals)
☐	☒	For null hypothesis testing, the test statistic (e.g. *F*, *t*, *r*) with confidence intervals, effect sizes, degrees of freedom and *P* value noted<br>*Give P values as exact values whenever suitable.*
☒	☐	For Bayesian analysis, information on the choice of priors and Markov chain Monte Carlo settings
☒	☐	For hierarchical and complex designs, identification of the appropriate level for tests and full reporting of outcomes
☒	☐	Estimates of effect sizes (e.g. Cohen's *d*, Pearson's *r*), indicating how they were calculated

Our web collection on statistics for biologists contains articles on many of the points above.

Software and code

Policy information about availability of computer code

Data collection	PhenoMaster data were collected by PhenoMaster TSE Systems and Home-cage CO2 measurements data were collected by nondispersive infrared (NDIR) CO2 sensor.
Data analysis	ImageJ (V1.53), GraphPad Prism (V9), R (V4.2.1) and Python (V3.11.0).

For manuscripts utilizing custom algorithms or software that are central to the research but not yet described in published literature, software must be made available to editors and reviewers. We strongly encourage code deposition in a community repository (e.g. GitHub). See the Nature Portfolio guidelines for submitting code & software for further information.

Data

Policy information about availability of data

All manuscripts must include a data availability statement. This statement should provide the following information, where applicable:

- Accession codes, unique identifiers, or web links for publicly available datasets
- A description of any restrictions on data availability
- For clinical datasets or third party data, please ensure that the statement adheres to our policy

The mass spectrometry proteomics data presented in Fig. 2 have been deposited in the Mass Spectrometry Interactive Virtual Environment (MassIVE) repository (https://massive.ucsd.edu/ProteoSAFe/static/massive.jsp) and are publicly available under accession code MSV000090693. All other data supporting this study are available within this Article and Supplemental Information. Source data are provided with this paper.

Human research participants

Policy information about studies involving human research participants and Sex and Gender in Research.

Reporting on sex and gender	This research did not involve human research participants.
Population characteristics	This research did not involve human research participants.
Recruitment	This research did not involve human research participants.
Ethics oversight	This research did not involve human research participants.

Note that full information on the approval of the study protocol must also be provided in the manuscript.

Field-specific reporting

Please select the one below that is the best fit for your research. If you are not sure, read the appropriate sections before making your selection.

☒ Life sciences ☐ Behavioural & social sciences ☐ Ecological, evolutionary & environmental sciences

For a reference copy of the document with all sections, see nature.com/documents/nr-reporting-summary-flat.pdf

Life sciences study design

All studies must disclose on these points even when the disclosure is negative.

Sample size	Sample size was chosen to ensure an adequate statistical power. For in vitro study, at least three biological replicates were used. The number of animal are described in the manuscript.
Data exclusions	No data exclusion.
Replication	All figure data was obtained in at least 3 successful independent sets of experiments.
Randomization	For in vitro studies, conditions were randomized into control and experimental conditions as described in each assay. All animals in our experiments were randomly allocated into different groups.
Blinding	In selected imaging experiments (where indicated in figure legends), quantification was performed with the investigator blinded to the identity of the samples.

Reporting for specific materials, systems and methods

We require information from authors about some types of materials, experimental systems and methods used in many studies. Here, indicate whether each material, system or method listed is relevant to your study. If you are not sure if a list item applies to your research, read the appropriate section before selecting a response.

Materials & experimental systems

n/a	Involved in the study	
☐	☒	Antibodies
☐	☒	Eukaryotic cell lines
☒	☐	Palaeontology and archaeology
☐	☒	Animals and other organisms
☒	☐	Clinical data
☒	☐	Dual use research of concern

Methods

n/a	Involved in the study	
☒	☐	ChIP-seq
☒	☐	Flow cytometry
☒	☐	MRI-based neuroimaging

Antibodies

Antibodies used	Rabbit polyclonal anti-TMEM135 Aviva Systems Biology Cat# ARP49773_P050; RRID:AB_2048451, WB (1:1000); IF (1:100)<br>Rabbit polyclonal anti-TMEM135 Novus Cat# NBP2-55549; RRID: N/A, WB (1:1000)<br>Rabbit polyclonal anti-Drp1 Cell Signaling Technology Cat# 8570; RRID:AB_10950498, WB (1:1000); IF (1:50)<br>Mouse monoclonal anti-COX4 Cell Signaling Technology Cat# 11967; RRID:AB_2797784, WB (1:1000), clone 4D11-B3-E8<br>Rabbit polyclonal anti-TOM20 Cell Signaling Technology Cat# 42406; RRID:AB_2687663,WB (1:1000)

Rabbit polyclonal anti-OPA1 Proteintech Cat# 27733-1-AP; RRID:AB_2810292, WB (1:1000)
Rabbit polyclonal anti-Phospho-Drp1 (Ser637) Cell Signaling Technology Cat# 4867; RRID:AB_10622027, WB (1:1000)
Rabbit polyclonal anti-Actin Cell Signaling Technology Cat# 8457; RRID:AB_10950489, WB (1:1000)
Rabbit polyclonal anti-PMP70 Sigma-Aldrich Cat# SAB4200181; RRID:AB_10639362, WB (1:1000)
Rabbit polyclonal anti-AKT Cell Signaling Technology Cat# 9272; RRID: AB_329827, WB (1:1000)
Rabbit polyclonal anti-Phospho-Akt (Ser473) Cell Signaling Technology Cat# 9271; RRID: AB_329825, WB (1:1000)
Rabbit polyclonal anti-Pex16 Proteintech Cat# 14816-1-AP; RRID:AB_2162250, WB (1:1000)
Rabbit polyclonal anti-PMP70-Atto-488 Sigma-Aldrich Cat# P0090; RRID:AB_1841112, IF (1:100)
Rabbit polyclonal anti-Pex19 Proteintech Cat# 14713-1-AP; RRID:AB_2162265,WB (1:1000)
Rabbit polyclonal anti-Pex14 Proteintech Cat# 10594-1-AP;RRID: AB_2252194 ,IF (1:50)
Rabbit monoclonal anti-PDI Cell Signaling Technology Cat# 3501; RRID:AB_2156433, WB (1:1000), clone C81H6
Rat monoclonal anti-LAMP2 Abcam Cat# ab13524; RRID: AB_2134736 ,WB (1:1000), clone GL2A7
Rabbit polyclonal anti-Lamin A/C Cell Signaling Technology Cat# 2032; RRID:AB_2136278, WB (1:1000)
Peroxidase IgG Fraction Monoclonal Mouse
Anti-Rabbit IgG, light chain specific Jackson Immunoresearch Cat# 211-032-171; RRID: AB_2339149, WB (1:2000)
Peroxidase AffiniPure Goat Anti-Mouse IgG, light
chain specific Jackson Immunoresearch Cat# 115-035-174; RRID: AB_2338512, WB (1:2000)
Goat anti-Rabbit IgG (H+L) Cross-Adsorbed Secondary Antibody, Alexa Fluor 594 Thermo Fisher Cat# A-11012; RRID:AB_141359, IF (1:500)
Goat anti-mouse IgG (H+L) Cross-Adsorbed Secondary Antibody, Alexa Fluor 488 Thermo Fisher Cat# A-11001; RRID:AB_2534069, IF (1:500)

Validation

TMEM135 (https://www.avivasysbio.com/tmem135-antibody-n-terminal-region-arp49773-p050.html)
Reactivity: Human, Mouse, Rat, Cow, Dog, Guinea Pig, Horse, Pig, Rabbit
Applications: WB, IHC
TMEM135 (https://www.novusbio.com/products/tmem135-antibody_nbp1-59377)
Reactivity: Human
Applications: WB, IHC
Drp1 (https://www.cellsignal.com/products/primary-antibodies/drp1-d6c7-rabbit-mab/8570?_requestid=214259)
Reactivity: Human, Mouse, Rat, Monkey
Applications: WB, IP, IF
COX4 (https://www.cellsignal.com/products/primary-antibodies/cox-iv-4d11-b3-e8-mouse-mab/11967)
Reactivity: Reactivity: Rat
Applications: Human, Mouse, Rat, Monkey
Applications: WB, IP, IHC, IF
TOM20 (https://www.cellsignal.com/products/primary-antibodies/tom20-d8t4n-rabbit-mab/42406)
Reactivity: Human, Mouse, Rat, Monkey
Applications: WB, IP, IHC, IF
OPA1(https://www.ptglab.com/products/OPA1-Antibody-27733-1-AP.htm)
Reactivity: Human, Mouse, Rat
Applications: WB, IP, IHC
Phospho-Drp1 (Ser637) (https://www.cellsignal.com/products/primary-antibodies/phospho-drp1-ser637-antibody/4867)
Reactivity: Rat
Applications: WB, IP
Actin (https://www.cellsignal.com/products/primary-antibodies/b-actin-d6a8-rabbit-mab/8457)
Reactivity: Human, Mouse, Rat, Monkey, D. melanogaster, Zebrafish
Applications: WB, IF, FC
PMP70 (https://www.sigmaaldrich.com/US/en/product/sigma/sab4200181)
Reactivity: Human, Mouse, Rat
Applications: WB, IF
AKT (https://www.cellsignal.com/products/primary-antibodies/akt-antibody/9272)
Reactivity: Human, Mouse, Rat, Hamster, Monkey, Chicken, D. melanogaster, Bovine, Dog, Pig, Guinea Pig
Applications: WB, ,IP, IF, FC
Phospho-Akt (Ser473) (https://www.cellsignal.com/products/primary-antibodies/phospho-akt-ser473-antibody/9271)
Reactivity: Human, Mouse, Rat, Hamster, Monkey, D. melanogaster, Bovine, Dog
Applications: WB, ,IP, IF, FC
Pex16 (https://www.ptglab.com/products/PEX16-Antibody-14816-1-AP.htm)
Reactivity: Human, Mouse, Rat
Applications: WB, IF
PMP70-Atto-488 (https://www.sigmaaldrich.com/US/en/product/sigma/p0090)
Reactivity: Human, Mouse, Rat
Applications: IF
Pex19 (https://www.ptglab.com/products/PEX19-Antibody-14713-1-AP.htm)
Reactivity: Human, Mouse
Applications: WB, IP, IHC
Pex14 (https://www.ptglab.com/products/PEX14-Antibody-10594-1-AP.htm)
Reactivity: Human, Mouse, Rat
Applications: WB, IP, IHC, IF, FC, ELISA
PDI (https://www.cellsignal.com/products/primary-antibodies/pdi-c81h6-rabbit-mab/3501)
Reactivity: Human, Mouse, Rat, Monkey
Applications: WB, IHC, IF
LAMP2 (https://www.abcam.com/products/primary-antibodies/lamp2-antibody-gl2a7-ab13524.html)
Reactivity: Human, Mouse, Rabbit
Applications: IHC, IF, IP, FC, WB
Lamin A/C (https://www.cellsignal.com/products/primary-antibodies/lamin-a-c-antibody/2032)

Reactivity: Human, Mouse, Rat
Applications: IHC, WB
Rabbit IgG, light chain specific Jackson Immunoresearch (https://www.jacksonimmuno.com/catalog/products/211-032-171)
Mouse IgG, light chain specific Jackson Immunoresearch (https://www.jacksonimmuno.com/catalog/products/115-035-174)
Goat anti-Rabbit IgG (H+L) Cross-Adsorbed Secondary Antibody, Alexa Fluor 594 (https://www.thermofisher.com/antibody/product/Goat-anti-Rabbit-IgG-H-L-Cross-Adsorbed-Secondary-Antibody-Polyclonal/A-11012)
Goat anti-mouse IgG (H+L) Cross-Adsorbed Secondary Antibody, Alexa Fluor 488 (https://www.thermofisher.com/antibody/product/Goat-anti-Mouse-IgG-H-L-Cross-Adsorbed-Secondary-Antibody-Polyclonal/A-11001)

Eukaryotic cell lines

Policy information about cell lines and Sex and Gender in Research

Cell line source(s)	Immortalized mouse brown preadipocytes were isolated in the Lodhi lab and have been previously described. Immortalized human brown preadipocytes were generated in the Tseng lab and have been previously described.
Authentication	We did not make efforts to authenticate cell lines.
Mycoplasma contamination	All cell lines were tested negative for mycoplasma.
Commonly misidentified lines (See ICLAC register)	No commonly misidentified cell were studied.

Animals and other research organisms

Policy information about studies involving animals; ARRIVE guidelines recommended for reporting animal research, and Sex and Gender in Research

Laboratory animals	Pex16-AKO, TMEM135-AKO, Adiponectin-Cre, TMEM135TG and Pex16-AKO-TMEM135TG mice were used in experiments starting at 8 to 10 weeks of age. These mice were generated on the C57BL/6J genetic background.Mice were maintained under constant temperature (22-24°C), circulating air and humidity (45-65%) with 12h:12h light/dark cycle. Mice had free access to food and water.
Wild animals	No wild animals were used in this study.
Reporting on sex	Both male and female animals were studied for metabolic phenotyping studies, as indicated in figure legends.
Field-collected samples	No field collected samples were used in the study.
Ethics oversight	All animal protocols were approved by the Washington University Institutional Animal Care and Use Committee.

Note that full information on the approval of the study protocol must also be provided in the manuscript.
